# Predictors of mortality in patients with hereditary hemorrhagic telangiectasia

**DOI:** 10.1186/s13023-020-01579-2

**Published:** 2021-01-06

**Authors:** K. P. Thompson, J. Nelson, H. Kim, L. Pawlikowska, D. A. Marchuk, M. T. Lawton, Marie E. Faughnan, the Brain Vascular Malformation Consortium HHT Investigator Group, Murali Chakinala, Marianne S. Clancy, Marie E. Faughnan, James R. Gossage, Katharine Henderson, Vivek Iyer, Raj S. Kasthuri, Helen Kim, Timo Krings, Michael T. Lawton, Doris Lin, Hans-Jurgen Mager, Douglas A. Marchuk, Justin P. McWilliams, Jamie McDonald, Ludmila Pawlikowska, Jeffrey Pollak, Felix Ratjen, Karen Swanson, Karel terBrugge, Dilini Vethanayagam, Andrew J. White, Pearce Wilcox

**Affiliations:** 1 Toronto HHT Centre, Li Ka Shing Knowledge Institute, St. Michael’s Hospital, 30 Bond St, Toronto, ON M5B-1W8 USA; 2 Division of Respirology, Department of Medicine, University of Toronto, Toronto, Canada; 3 Department of Anesthesia and Perioperative Care, Center for Cerebrovascular Research, University of California, San Francisco, CA USA; 4 Institute for Human Genetics, University of California, San Francisco, CA USA; 5 Department of Epidemiology and Biostatistics, University of California, San Francisco, CA USA; 6 Department of Molecular Genetics and Microbiology, Duke University Medical Center, Durham, NC USA; 7 Barrow Neurological Institute, Phoenix, AZ USA

**Keywords:** Hereditary hemorrhagic telangiectasia, Vascular malformation, Arteriovenous malformation, Telangiectasia, Predictors of mortality

## Abstract

**Background:**

Retrospective questionnaire and healthcare administrative data suggest reduced life expectancy in untreated hereditary hemorrhagic telangiectasia (HHT). Prospective data suggests similar mortality, to the general population, in Denmark’s centre-treated HHT patients. However, clinical phenotypes vary widely in HHT, likely affecting mortality. We aimed to measure predictors of mortality among centre-treated HHT patients. HHT patients were recruited at 14 HHT centres of the Brain Vascular Malformation Consortium (BVMC) since 2010 and followed annually. Vital status, organ vascular malformations (VMs) and clinical symptoms data were collected at baseline and during follow-up (N = 1286). We tested whether organ VMs, HHT symptoms and HHT genes were associated with increased mortality using Cox regression analysis, adjusting for patient age, sex, and smoking status.

**Results:**

59 deaths occurred over average follow-up time of 3.4 years (max 8.6 years). A history of anemia was associated with increased mortality (HR = 2.93, 95% CI 1.37–6.26, *p* = 0.006), as were gastro-intestinal (GI) bleeding (HR = 2.63, 95% CI 1.46–4.74, *p* = 0.001), and symptomatic liver VMs (HR = 2.10, 95% CI 1.15–3.84, *p* = 0.015). Brain VMs and pulmonary arteriovenous malformations (AVMs) were not associated with mortality (*p* > 0.05). Patients with *SMAD4* mutation had significantly higher mortality (HR = 18.36, 95% CI 5.60–60.20, *p* < 0.001) compared to patients with *ACVRL1* or *ENG* mutation, but this estimate is imprecise given the rarity of *SMAD4* patients (n = 33, 4 deaths).

**Conclusions:**

Chronic GI bleeding, anemia and symptomatic liver VMs are associated with increased mortality in HHT patients, independent of age, and in keeping with the limited treatment options for these aspects of HHT. Conversely, mortality does not appear to be associated with pulmonary AVMs or brain VMs, for which patients are routinely screened and treated preventatively at HHT Centres. This demonstrates the need for development of new therapies to treat chronic anemia, GI bleeding, and symptomatic liver VMs in order to reduce mortality among HHT patients.

## Background

Hereditary hemorrhagic telangiectasia (HHT) is a rare dominant genetic disorder with an estimated prevalence of approximately 1 in 5000–10,000 [1–5], affecting children and adults, often involving multiple organs. HHT is characterized by the presence of vascular malformations (VMs), including arteriovenous malformations (AVMs) of the lung, liver, brain, spinal cord and smaller mucosal lesions (telangiectasia) of the nose, mouth and gastro-intestinal (GI) tract [6, 7]. These lesions lead to acute life-threatening bleeding, stroke, heart failure and death, as well as chronic bleeding from the nose and GI tract [8–12]. In other words, most HHT patients suffer from daily symptoms and are at risk of life-threatening complications. To date, there are no highly effective therapies to manage the chronic symptoms and only limited preventative management of serious complications. As novel therapies for HHT now become available, we need a paradigm shift in HHT research to be able to test these therapies and bring them to the patients that need them most. There is an urgent need therefore to understand mortality and other severe outcomes in HHT and their predictors.

Mortality and its predictors remain poorly understood in HHT to date. Retrospective questionnaire-based studies [13–15] reported reduced median life expectancy of affected parents of HHT patients, with untreated disease, compared to non-HHT parents [13, 14] and to a general population in Germany [15]. These studies primarily reflect the care of HHT patients prior to HHT-centre based care, which has largely evolved over the last 20 years and has become more standardized since the publication of International HHT Guidelines [6]. One group [13] reported no difference in life-expectancy by HHT gene mutated, the other [14] reporting worse life-expectancy in patients with *ENG* mutation, with the largest difference in women. We speculate that this difference was related to complications of untreated lung AVMs and brain VMs, common in *ENG* mutation carriers, before the HHT centre-based care era of screening and treatment.

Administrative data also suggests reduced survival in HHT patients, compared to controls, in the UK [11], with a hazard ratio for death of 2.0 (CI 1.6–2.6) in HHT cases compared to controls, and a median age at death 3 years younger. However, this was based on historical data (1986–2011) from primary care practice, and therefore again likely that these results primarily reflect the routine care of HHT patients rather than specialized centre-based care, and also mostly prior to dissemination of the 2009 International HHT Guidelines [6]. In other words, likely few patients received preventative management for lung and brain AVMs.

The only prospective survival study is small, with 75 Danish patients followed for 20 years at one center and showed similar survival to controls [16], suggesting that patients treated at Specialized HHT Centres with routine preventative management may have better outcomes. Recent reports using US Nationwide Inpatient Sample (NIS) administrative data from 2000 to 2012 [17, 18] support this point, as patients with HHT hospitalized at high-volume centers (> 8 HHT patients discharged per year) had better outcomes, with significantly lower in-hospital mortality (1.2% vs 2.4%; *p* < 0.001) and higher home discharge rates (78.1% vs 71.6%; *p* < 0.001), compared to low-volume centres.

Few studies reported to date have detailed cause of death and none have identified predictors of mortality. This is particularly relevant in a multi-system disease such as HHT, where clinical heterogeneity is the rule, with widely variable clinical phenotypes. We aimed to measure predictors of mortality among centre-treated HHT patients.

## Methods

*Cohort* The study includes 1286 HHT patients enrolled by the Brain Vascular Malformation Consortium (BVMC) at multiple recruiting centers in the US, Canada and the Netherlands between 2010 and 2018 with at least one follow-up visit or verification of death. Cohort recruitment has been previously described [12, 19]. All patients provided written informed consent. The study protocol was approved by the institutional review board at each recruiting centre. Patients were screened for organ VMs and other clinical features according to standard clinical practice and International HHT Guidelines [6], including: comprehensive history, physical, routine blood tests, screening for pulmonary AVM by contrast echocardiography, brain VM screening by magnetic resonance imaging, clinical screening for liver VM (chronic right upper quadrant pain, portal hypertension, high-output heart failure, liver bruit on examination, abnormal liver function tests) and clinical screening for recurrent spontaneous epistaxis (> 1 episode per month for > 1 year), and HHT-related GI-bleeding (anemia, iron deficiency, known GI telangiectases on endoscopy, melena, rectal bleeding). As part of their routine clinical care, if screening was positive for pulmonary AVM or brain VM, patients underwent further diagnostic imaging and treatment, where appropriate. If clinical assessment was suggestive of symptomatic liver VM, diagnostic imaging was recommended and therapy where appropriate. If initial clinical assessment was suggestive of HHT-related GI bleeding, then diagnostic endoscopy was recommended, and endoscopic, medical and supportive therapies were undertaken on a case-by-case basis. The BVMC HHT cohort targets 25% brain VM-positive patients; other characteristics are similar to other cohorts [20, 21].

*Analysis* Vital status, organ VMs, genetics and clinical data were collected at baseline. We tested whether organ VMs, HHT symptoms and HHT genes were individually associated with increased mortality using Cox proportional hazards regression analysis, adjusting for patient age (modeled as a categorical variable: 0–19, 20–39, 40–59, or 60+ years old), sex, and smoking history (current or past smoker versus never smoker). Additionally, we used a backward elimination procedure, keeping in patient age, sex, and smoking history, to determine which symptoms contributed to a multivariable regression model. For this procedure, we sequentially removed predictors with the largest *p* value until all included predictors had a *p* value less than 0.05. Since some clinical data was missing, we re-ran all Cox regression analyses using 10 imputation data sets created using the chained equations imputation technique. Finally, we generated Kaplan–Meier curves stratified by various groupings. Cox regression results are reported as hazard ratios (HR) with 95% confidence intervals (CI). *P* values less than 0.05 were considered significant. Stata 15.1 was used to perform statistical analyses (StataCorp. 2017. *Stata Statistical Software: Release 15*. College Station, TX: StataCorp LLC.).

## Results

Patient characteristics are reported in Table 1, including demographics, HHT manifestations, and gene mutations.

**Table 1:** Demographic data, HHT manifestations, and gene mutations

Characteristics	Overall	Alive	Dead
Count	1286	1227	59
Age at enrollment, mean ± SD	46.0 ± 19.7	45.3 ± 19.5	60.8 ± 18.0
Age category			
< 20	171 (13%)	168 (14%)	3 (5%)
20–39	263 (20%)	258 (21%)	5 (8%)
40–59	511 (40%)	500 (41%)	11 (19%)
> 60	341 (27%)	301 (25%)	40 (68%)
Female	749 (58%)	718 (59%)	31 (53%)
Smoker (past or current)	428/1231 (35%)	395/1178 (34%)	33/53 (62%)
Brain VM	278 (22%)	268 (22%)	10 (17%)
Pulmonary AVM	615/1235 (50%)	585/1178 (50%)	30/57 (53%)
Symptomatic liver VM	217/1217 (18%)	196/1162 (17%)	21/55 (38%)
GI bleeding	216/1233 (18%)	189/1177 (16%)	27/56 (48%)
Anemia	591/1234 (48%)	550/1181 (47%)	41/53 (77%)
Genotype			
ACVRL1	357/886 (40%)	343/854 (40%)	14/32 (44%)
ENG	496/886 (56%)	482/854 (56%)	14/32 (44%)
SMAD4	33/886 (4%)	29 (3%)	4/32 (13%)

Values are n (%) or n/total (%), unless otherwise specified

59 deaths occurred, of 1286 patients, during mean follow-up time of 3.4 years (maximum follow-up time was 8.6 years with total 4411 patient-years of follow-up). Cox regression analyses results are summarized in Table 2. GI bleeding was associated with increased mortality (HR = 2.63, 95% CI 1.46–4.74, *p* = 0.001), as were symptomatic liver VMs (HR = 2.10, 95% CI 1.15–3.84, *p* = 0.015). History of anemia was also associated with increased mortality (HR = 2.93, 95% CI 1.37–6.26, *p* = 0.006). Note that GI bleeding, liver VMs, and history of anemia were associated with one another at baseline (Fisher’s exact test *p* < 0.001 for all three possible pairings). Brain VMs and pulmonary AVMs were not significantly associated with mortality (*p* > 0.05). Patients with *SMAD4* mutation had significantly higher mortality (HR = 18.36, 95% CI 5.60–60.20, *p* < 0.001) compared to patients with *ACVRL1/ENG* mutations, but the estimate is imprecise given the rarity of *SMAD4* patients (n = 33, 4 deaths). There was no significant difference in mortality between patients with *ACVRL1* and *ENG* mutation (*p* > 0.05). The backward elimination procedure identified a model that included both anemia (HR = 2.44, 95% CI 1.10–5.42, *p* = 0.028) and GI bleeding (HR = 2.25, 95% CI 1.21–4.18, *p* = 0.010). Cox regression analyses of each characteristic individually using imputed data largely agreed with the complete-case analyses and are presented in the Additional file 1: Table S1. Performing the backward elimination procedure on the imputed data retained only GI bleeding as a significant predictor (HR = 2.77, 95% CI 1.60–4.82, *p* < 0.001).

**Table 2:** Cox regression results of HHT manifestations and gene mutations

Characteristic	HR	95% CI	*p* value
Brain VM	0.76	(0.35, 1.66)	0.494
Pulmonary AVM	1.12	(0.64, 1.96)	0.681
Symptomatic Liver VM	2.10	(1.15, 3.84)	0.015
GI bleeding	2.63	(1.46, 4.74)	0.001
Anemia	2.93	(1.37, 6.26)	0.006
ACVRL1 (vs. ENG)	1.54	(0.70, 3.38)	0.286
SMAD4 (vs. ACVRL1/ENG)	18.36	(5.60, 60.20)	< 0.001

Characteristics were tested individually, while adjusting for age, sex, and smoking status

**Fig. 1:**
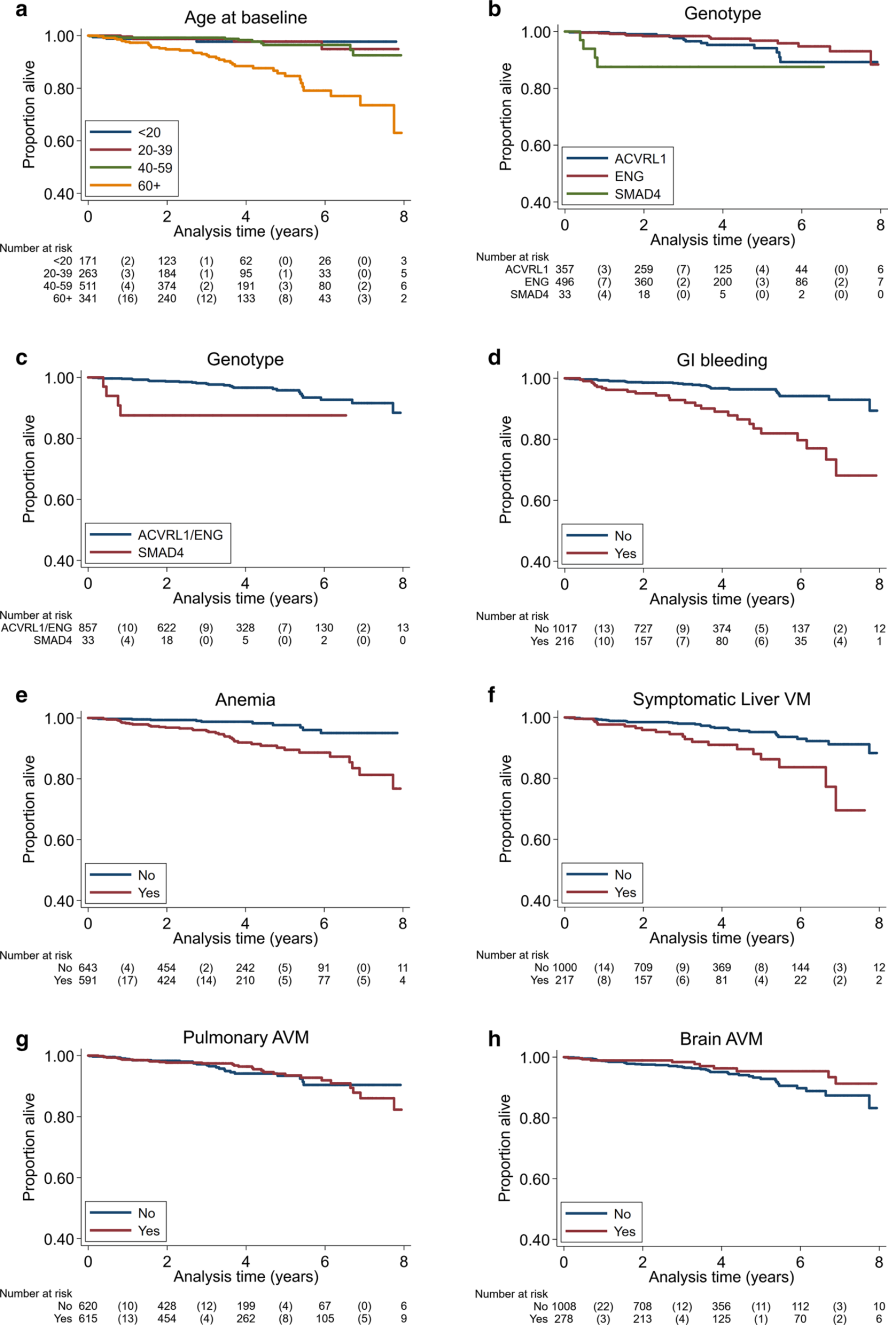
Kaplan–Meier survival curves for age (**a**), genotype (**b**, **c**), GI bleeding (**d**), anemia (**e**), symptomatic liver VM (**f**), pulmonary AVM (**g**), and brain VM (**h**)

Kaplan–Meier survival curves are displayed in Fig. 1, for age, symptomatic liver VM, anemia, GI bleeding, pulmonary AVM, brain VM and genotype (*SMAD4* vs *ACVRL1/ENG*).

### Cause of death

Detailed chart review for cause of death was available in 46 patients: 23 died from HHT or JP-HHT related complications; 23 died from non-HHT causes. Cause of death was unavailable or unknown in the other 13 patients. In the 23 patients who died from HHT or JP-HHT related complications, specific causes included heart failure from liver VMs (6), chronic severe HHT-related bleeding (7), brain abscess (2, including one with diffuse pulmonary AVM), staph infections (2), pulmonary hemorrhage (1, in a SMAD4 patient with JP-HHT and diffuse pulmonary AVMs), pulmonary hypertension (2), acute rejection post-liver transplant for liver VMs (1), intracranial hemorrhage (1) and metastatic colon cancer (in a SMAD4 patient with HHT, at the age of 29). Of the 23 deaths from non-HHT causes, specific causes included cancer (7), accident (3), pulmonary fibrosis (2), myocardial infarction (1), congestive heart failure (1), pneumonia (1), COPD (1), dementia (1), stroke (1), hepatitis C (1), venous thromboembolism (1) and “natural causes” (3).

## Discussion

In this large, multi-center prospective study of HHT patients cared for in specialized HHT centers, we demonstrate that HHT-related GI bleeding, anemia and symptomatic liver VMs are predictors of mortality. This is in keeping with the limited treatment options for these aspects of HHT and highlights the importance of development and trials for new therapies for these aspects of HHT.

We demonstrate here that GI bleeding and anemia are predictors of mortality, independent of age, in both the univariate and multivariable models. GI bleeding is also shown to be a predictor of mortality in the imputed data model. This is despite specialized center care according to HHT guidelines, typically including supportive management of anemia and also endoscopic management of clinically evident GI bleeding, with consideration for additional agents such as tranexamic acid or other medical therapies, though this management data was not collected. These results are in keeping with results from an earlier cohort at the Toronto HHT Centre, reporting reduced survival in HHT patients with GI bleeding, in abstract form [22]. Others have demonstrated that GI bleeding is a frequent cause for emergency room visits and hospitalization amongst HHT patients [8, 18] and patients with GI involvement require more blood transfusions, emergency room visits and hospital admissions compared to other HHT patients [23], all supporting the significant morbidity associated with this complication of HHT. In our series, several deaths were the result of chronic severe bleeding. The morbidity and mortality associated with HHT-related GI bleeding suggests an urgent need for the development of new therapies for GI bleeding and anemia. Systemic therapies are of particular interest, given that GI bleeding typically occurs from a large number of small lesions [24], and supported by recent promising reports of intravenous bevacizumab [25] and oral pazopanib [26] for management of severe GI bleeding, as well as chronic epistaxis.

We also demonstrate that the presence of symptomatic liver VMs is a predictor of mortality in the univariate model, though it did not remain a significant predictor in the multivariable model likely due to overlap with GI bleeding, given that both are associated with *ACVRL1* mutation [27]. The association between liver VMs and mortality was observed despite specialized center care for liver VMs according to HHT guidelines [6] and its complications, typically including supportive and medical management of high-output heart failure, portal hypertension, and biliary ischemia, and consideration for liver transplantation and intravenous bevacizumab in severe and refractory cases, though this management data was not collected. These results are in keeping with an earlier report of significant morbidity and mortality from liver VMs in HHT patients, with 25% of liver VM patients experiencing complications and 5% death from liver VM-related complications [28] over a median 44 months of follow-up. In our series, liver VMs were the most common cause of death. Given the demonstrated morbidity and mortality associated with liver VMs, once again there is an urgent need for new therapies. Successful liver transplantation for liver VMs has been reported [29], as has management with intravenous bevacizumab [30, 31], though these are limited and expensive options, considered only presently for those with severe and symptomatic liver VMs. Again, novel systemic therapies would potentially have the particular advantage of simultaneously addressing the riskiest aspects of HHT: liver VMs and chronic GI bleeding.

Brain VMs and pulmonary AVMs are not predictors of mortality in center-based care HHT patients reported here. We speculate that this result is due to current routine screening and preventative treatment of pulmonary AVMs in specialized HHT Centres, as well as screening and preventative treatment of brain VMs in selected HHT patients. Although 3 of the documented deaths in our study were related to pulmonary AVM complications, 2 of these 3 deaths occurred in patients with diffuse pulmonary AVMs. Patients with diffuse pulmonary AVMs are a rare severe subset (5%) of the patients with pulmonary AVMs, with significant morbidity, including high risk of neurologic complications, and mortality [32, 33]. In other words, though diffuse pulmonary AVMs were an important cause of death in our series, overall the presence of pulmonary AVMs was not associated with mortality. This underlines the importance of developing therapies for patients with the rare form of diffuse pulmonary AVMs; it also supports the current practice of preventative screening and management of the typical pulmonary AVMs, as per HHT Guidelines [6], and the recent recommendation by the European Reference Network for Rare Vascular Diseases (VASCERN) proposing pulmonary AVM screening as one of five metrics to identify healthcare providers of good care [34].

We observed that *SMAD4* mutation is associated with higher mortality compared to *ACVRL1* or *ENG* mutation in the univariate and imputed data models. However, this estimate is imprecise given the small number of *SMAD4* patients. HHT patients with *SMAD4* mutation typically have an overlap syndrome with Juvenile Polyposis (JP), with patients developing both JP and HHT phenotypes [35, 36], with risk of early-onset gastrointestinal cancer and associated mortality [37, 38] In other words, the JP-associated risk of colorectal cancer in patients with *SMAD4* mutation may explain the observed mortality association with *SMAD4* in this study.

The findings of this study should be considered in light of some potential limitations. First, there were some missing data regarding organ involvement and detailed cause of death. However secondary survival analyses using imputed data supported the association of GI bleeding with mortality. Second, information regarding severity of epistaxis was not available and so epistaxis severity could not be studied as a predictor of HHT mortality or in relation to anemia. Similarly, information on the severity of liver VMs, brain VMs, and pulmonary AVMs, as well as the lesional burden of GI telangiectasia, was not available for this study, rather the analyses were performed based on their presence or absence. This may have led us to underestimate the association between organ VMs and mortality. In other words, this may have limited our power to detect a mortality association for brain VMs, for example. However, despite this limitation, we detected significant associations for liver VMs, GI bleeding and anemia, supporting the significance of these results. Information on mediations that might influence mortality and bleeding, such as anti-platelet and anticoagulant medications was not available and therefore not adjusted for in the analyses. Another potential limitation of this study is survivor bias, particularly relevant to brain VMs, given brain VMs can cause mortality in children with HHT [39], and this may have prevented us from detecting an association with mortality. Finally, we did not collect detailed data on clinical management of patient’s organ VMs, though all participating centers report following standard practice according to the 2009 International HHT Guidelines.

## Conclusions

Chronic GI bleeding, anemia, and symptomatic liver VMs are associated with increased mortality in HHT patients, independent of age, and in keeping with the limited treatment options available for these aspects of HHT. Conversely, mortality does not appear to be associated with pulmonary AVMs or brain VMs, for which patients are routinely screened and treated preventatively at HHT Centers. This demonstrates the urgent need for development of new therapies to preventatively treat chronic GI bleeding, anemia, and symptomatic liver VMs in order to reduce mortality among HHT patients.

## Supplementary information

**Additional file 1: Table S1.** Imputed Cox regression results of HHT manifestations and gene mutations.

## Data Availability

All data generated or analyzed during this study are included in this published article and its supplementary information files.
