# Supplementary material for: Predictors of mortality in patients with hereditary hemorrhagic telangiectasia
Source: Orphanet J Rare Dis. 2021 Jan 6;16:12. doi: 10.1186/s13023-020-01579-2 (PMC7789194; doi:10.1186/s13023-020-01579-2)
Supplement: Supplementary file 1 — Additional file 1: Table S1. Imputed Cox regression results of HHT manifestations and gene mutations. [file 13023_2020_1579_MOESM1_ESM.docx]

| **Table S1: Imputed Cox regression results of HHT manifestations and gene mutations.** | | | |
| --- | --- | --- | --- |
| Characteristics were tested individually, while adjusting for age, sex, and smoking status. | | | |
| Characteristic | HR | 95% CI | p-value |
| Brain VM | 0.88 | (0.43, 1.77) | 0.711 |
| Pulmonary AVM | 1.14 | (0.67, 1.93) | 0.622 |
| Symptomatic Liver VM | 2.05 | (1.14, 3.68) | 0.016 |
| GI bleeding | 2.77 | (1.60, 4.82) | <0.001 |
| Anemia | 2.23 | (1.11, 4.49) | 0.005 |
| ACVRL1 (vs. ENG) | 1.27 | (0.61, 2.65) | 0.516 |
| SMAD4 (vs. ACVRL1/ENG) | 10.56 | (3.77, 29.59) | <0.001 |
